# Role of Lipidomics in Respiratory Tract Infections: A Systematic Review of Emerging Evidence

**DOI:** 10.3390/microorganisms13092190

**Published:** 2025-09-19

**Authors:** Vasiliki E. Georgakopoulou, Konstantinos Dodos, Vassiliki C. Pitiriga

**Affiliations:** 1 Department of Pathophysiology, Laiko General Hospital, National and Kapodistrian University of Athens, 11527 Athens, Greece; 2 Laboratory of Physiology, School of Medicine, Aristotle University of Thessaloniki, 54124 Thessaloniki, Greece; 3 Department of Microbiology, Medical School of Athens, National and Kapodistrian University of Athens, 75 Mikras Asias Street, 11527 Athens, Greece

**Keywords:** lipidomics, respiratory tract infections, phosphatidylcholines, sphingolipids, biomarkers, pneumonia

## Abstract

Lower respiratory tract infections (LRTIs) remain a major cause of global morbidity and mortality, yet accurate pathogen identification and risk stratification continue to pose clinical challenges. Lipidomics—the comprehensive analysis of lipid species within biological systems—has emerged as a promising tool to unravel host–pathogen interactions and reveal novel diagnostic and prognostic biomarkers. This systematic review synthesizes evidence from nine original studies applying mass spectrometry-based lipidomic profiling in human LRTIs, including community-acquired pneumonia (CAP), ventilator-associated pneumonia (VAP), and coronavirus disease 2019 (COVID-19). Across diverse study designs, sample types, and analytical platforms, consistent alterations in lipid metabolism were observed. Perturbations in phospholipid classes, particularly phosphatidylcholines (PCs) and lysophosphatidylcholines (LPCs), were frequently associated with disease severity and immune activation. The ratios of PC to LPC and phosphatidylethanolamine (PE) to lysophosphatidylethanolamine (LPE) emerged as markers of inflammatory remodeling. Sphingolipids—including sphingomyelins (SMs) and sphingosine-1-phosphate (S1P)—were identified as key modulators of monocyte and neutrophil activation. Fatty acid–derived lipid mediators such as oxylipins (e.g., 12,13-epoxyoctadecenoic acid and 15-hydroxyeicosatetraenoic acid) and acylcarnitines reflected pathogen-specific immune responses and mitochondrial dysfunction. Several lipid-based classifiers demonstrated superior diagnostic and prognostic performance compared to conventional clinical scores, including the CURB-65 and pneumonia severity index. However, significant heterogeneity in experimental design, lipid identification workflows, and reporting standards limits inter-study comparability. While preliminary findings support the integration of lipidomics into infectious disease research, larger multi-omic and longitudinal studies are required. This review provides the first comprehensive synthesis of lipidomic alterations in human LRTIs and highlights their emerging translational relevance.

## 1. Introduction

Lower respiratory tract infections (LRTIs) continue to pose a significant public health burden worldwide, encompassing a broad spectrum of conditions ranging from viral illnesses such as influenza, respiratory syncytial virus (RSV), and severe acute respiratory syndrome coronavirus 2 (SARS-CoV-2), to bacterial pneumonias caused by pathogens like *Streptococcus pneumoniae*, *Haemophilus influenzae*, and *Klebsiella pneumoniae* [1,2,3,4]. These infections can be classified based on their anatomical location—upper or lower respiratory tract—as well as by the setting in which they are acquired, distinguishing community-acquired pneumonia (CAP) from nosocomial infections such as hospital-acquired pneumonia (HAP) and ventilator-associated pneumonia (VAP) [5]. Despite advances in diagnostic microbiology and host biomarker discovery, challenges remain in early detection, pathogen-specific diagnosis, and prognostication of disease severity in both adult and pediatric populations.

These diagnostic and prognostic limitations are particularly critical given that LRTIs remain among the leading causes of global morbidity and mortality, disproportionately affecting young children, older adults, and immunocompromised patients [3]. Conventional diagnostic tools, such as cultures, antigen-based assays, or commonly used biomarkers including C-reactive protein (CRP) and procalcitonin (PCT), often lack sensitivity and specificity, delaying appropriate treatment decisions. Similarly, clinical severity scores such as the pneumonia severity index (PSI) and CURB-65 may not consistently predict outcomes across heterogeneous patient populations [5]. This highlights an urgent need for novel biomarkers and molecular tools that can improve early pathogen identification, refine risk stratification, and guide therapeutic interventions.

In recent years, the emergence of lipidomics as a subfield of metabolomics has offered new opportunities for understanding the pathophysiological basis of infectious diseases [6]. Lipidomics entails the comprehensive characterization of lipid species within biological systems using mass spectrometry–based approaches such as liquid chromatography–mass spectrometry (LC-MS), gas chromatography–mass spectrometry (GC-MS), and shotgun lipidomics [7]. These technologies allow the quantification of hundreds to thousands of lipid molecules, including glycerophospholipids, sphingolipids, fatty acids, and sterols, across various biological matrices [8,9,10]. By providing a systems-level overview of lipid remodeling during infection, lipidomics offers a unique opportunity to link clinical phenotypes with underlying host–pathogen biology, thereby enhancing both diagnostic precision and prognostic assessment in respiratory infections.

Lipidomics has become an increasingly valuable tool for studying host–pathogen interactions, given the essential roles of lipids in membrane structure, energy balance, and immune signaling [11]. Bioactive lipid mediators such as lysophosphatidylcholines (LPCs), sphingolipids, oxylipins, and bile acids are often dysregulated in LRTIs, potentially affecting immune responses and disease progression. Integrated omics studies have shown that plasma lipidomic profiles can differentiate viral from bacterial CAP, offering promising diagnostic insights [12]. Specific phosphatidylcholines (PCs) (e.g., PC 16:0_18:1, 36:4, 38:6), identified via ultra-high performance liquid chromatography–tandem mass spectrometry (UHPLC-MS/MS), have been associated with inflammation, oxygenation, and 30-day outcomes, outperforming clinical scores like pneumonia severity index (PSI) and CURB-65 in predicting severe CAP [13].

Evidence from RSV and SARS-CoV-2 studies further supports lipidomics’ diagnostic and prognostic potential. RSV-induced pneumonia alters lipid metabolism in lungs and plasma, especially affecting phospholipids and acylcarnitines [14]. In COVID-19 acute respiratory distress syndrome (ARDS), lipidomic analysis of tracheal aspirates revealed distinct lipid changes associated with VAP, with sphingomyelin (34:1) and PC (O-34:1) outperforming C-reactive protein (CRP) and procalcitonin (PCT) as biomarkers. Similarly, pediatric CAP studies have identified reduced LPCs and increased sphinganine, reflecting inflammation-related metabolic changes [15].

Notably, the clinical value of lipidomics has already been demonstrated in several other fields of medicine. In cardiovascular research, plasma lipid signatures have been integrated into risk prediction models for atherosclerotic disease and heart failure [8]. In oncology, lipidomics has contributed to cancer subtyping and treatment monitoring, particularly in breast and colorectal cancer [9]. In metabolic disorders, altered lipid pathways have been used to identify biomarkers of insulin resistance and non-alcoholic fatty liver disease [10]. These advances illustrate the translational potential of lipidomics and provide a compelling rationale for its application to respiratory tract infections, where reliable diagnostic and prognostic tools remain limited. Despite these promising findings, lipidomic applications in LRTIs remain underexplored relative to other ‘omics’ disciplines. Heterogeneity in lipidomic platforms, study designs, and clinical endpoints has limited cross-study comparability and translational relevance. Nonetheless, the growing body of literature suggests that lipid profiling may yield novel biomarkers for early diagnosis, disease stratification, and mechanistic understanding of LRTIs across diverse patient populations.

The aim of this systematic review is to synthesize existing evidence on the use of lipidomics in respiratory tract infections. Specifically, the review explores lipidomic alterations associated with various pathogens, the biological functions of dysregulated lipids in infection pathophysiology, and the diagnostic and prognostic potential of lipid-based biomarkers in both viral and bacterial LRTIs. Furthermore, this work identifies methodological gaps and provides recommendations for future studies aimed at integrating lipidomic tools into clinical infectious disease research.

## 2. Methods

This systematic review was designed in accordance with the Preferred Reporting Items for Systematic Reviews and Meta-Analyses (PRISMA) 2020 statement [16]. It has also been registered in the International Prospective Register of Systematic Reviews (PROSPERO) with ID number CRD420251103189.

### 2.1. Search Strategy

A systematic and comprehensive literature search was performed across four major electronic bibliographic databases—PubMed, Scopus, Web of Science, and Google Scholar—from their respective inceptions through August 2025. The search strategy was constructed using a combination of controlled vocabulary terms (e.g., Medical Subject Headings [MeSH]) and relevant free-text terms to maximize sensitivity and capture a broad spectrum of relevant studies.

The core search concepts included lipidomics and respiratory infections. Key search terms encompassed: “lipidomics”, “respiratory tract infection”, “community-acquired pneumonia”, “hospital-acquired pneumonia”, “ventilator-associated pneumonia”, “viral pneumonia”, “bacterial pneumonia”, “COVID-19”, “SARS-CoV-2”, “RSV”, “influenza”, “mass spectrometry”, “tuberculosis” “ultra-high-performance liquid chromatography”, “liquid chromatography–mass spectrometry”, “gas chromatography–mass spectrometry” “sphingolipids”, “phospholipids”, “oxylipins”, “lysophospholipids”, “bronchoalveolar lavage”, “tracheal aspirate”, “serum”, and “plasma”.

Boolean operators (AND, OR), truncation symbols, and field-specific tags (e.g., [MeSH Terms], [Title/Abstract]) were utilized to enhance the precision and breadth of the search. The full search syntax was adapted to the indexing system of each database. Additionally, the reference lists of all included full-text articles were manually screened to identify potentially eligible studies not captured by electronic search.

### 2.2. Inclusion and Exclusion Criteria

Studies were eligible for inclusion if they employed any form of lipidomic analysis, whether targeted or untargeted, and presented original research data relevant to LRTIs. Eligible study designs included both observational studies, such as cohort, case–control, and cross-sectional studies, and interventional studies, including clinical trials or controlled experimental models. The review considered studies involving human participants (patients or healthy controls), provided the respiratory infection was clearly defined and lipidomic data were reported. Acceptable sample types included serum, plasma, sputum, bronchoalveolar lavage fluid, tracheal aspirates, or other biologically relevant specimens collected in the context of LRTIs. To be included, studies had to utilize recognized lipidomic technologies such as LC-MS, GC-MS, nuclear magnetic resonance spectroscopy (NMR), or shotgun lipidomics, and report specific findings on lipid species, lipid class alterations, or lipid pathway perturbations linked to the infection.

Studies were excluded if they did not involve LRTIs or if lipidomic analysis was not performed. Publications that focused solely on other omics approaches (e.g., proteomics or transcriptomics) without presenting lipid-specific results were not considered eligible. Secondary literature including reviews, editorials, commentaries, expert opinions, and methodological papers without primary lipidomic data were also excluded. Additionally, case reports, small case series with fewer than five patients, animal studies and conference abstracts that lacked sufficient information on methods or results were not included. Only studies published in English and accessible in full-text format were considered for final inclusion.

### 2.3. PRISMA Process

The literature search yielded a total of 2438 records across four major databases: PubMed (782), Scopus (641), Web of Science (598), and Google Scholar (417). An additional 27 articles were identified through manual screening of reference lists from relevant reviews and included studies, bringing the total to 2465 records.

After the removal of 715 duplicate entries, 1750 unique records remained for title and abstract screening. This initial screening was conducted independently by two reviewers, resulting in the exclusion of 1555 records that did not meet the inclusion criteria. These excluded records primarily focused on unrelated diseases, lacked lipidomic analysis, or were review articles, commentaries, or editorials.

A total of 195 full-text articles were retrieved and assessed for eligibility. During full-text screening, 179 articles were excluded for the following reasons: 46 studies lacked a lipidomic component, 43 did not pertain to respiratory infections or tuberculosis, 26 were reviews or methodological articles, 23 were conference abstracts without sufficient data, 19 were published in languages other than English, 16 did not report original research or quantitative lipid data relevant to LRTIs/tuberculosis (TB), and 6 were animal studies.

Following this selection process, 16 studies met all predefined inclusion criteria and were included in the final qualitative synthesis. These encompassed diverse respiratory pathogens, including community-acquired pneumonia, COVID-19, ventilator-associated pneumonia, *Mycoplasma pneumoniae* infection, and tuberculosis.

**Figure 1:**
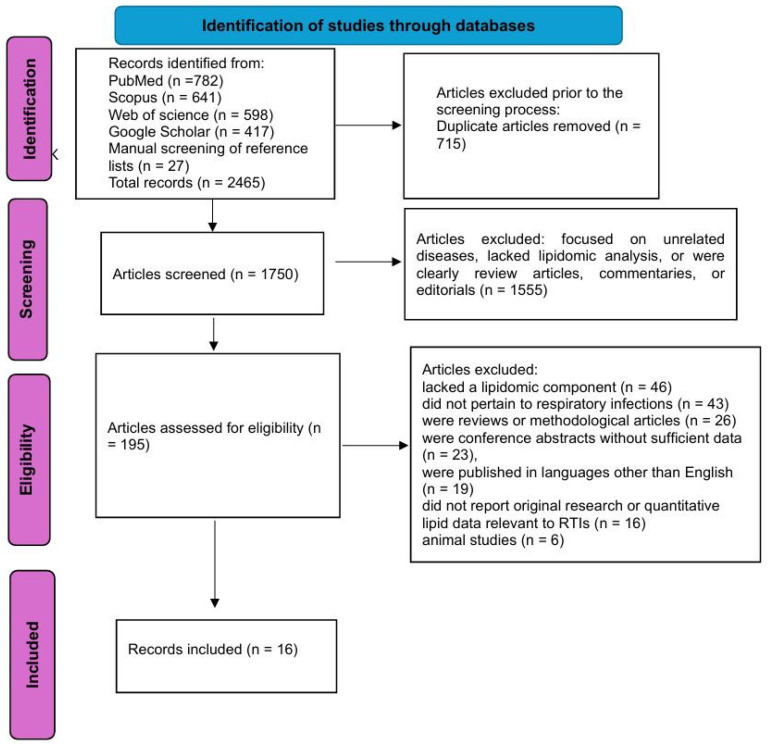
The flowchart of the study selection process.

Figure 1 illustrates the study selection process.

### 2.4. Quality Assessment

The methodological quality of the included studies was assessed using the Newcastle–Ottawa Scale (NOS), a validated tool specifically designed for evaluating the risk of bias in non-randomized observational studies, including cohort and case–control designs [17].

### 2.5. Data Extraction

A standardized data extraction form was used to record study characteristics, including author, year of publication, country, study design, infection type, sample source, analytical platform (e.g., LC-MS, GC-MS), number and classes of lipid species identified, and major lipidomic findings. Outcomes of interest included the identification of specific lipids associated with infection, the use of lipidomics for differential diagnosis or prognosis, and mechanistic insights into host–pathogen interactions mediated by lipids.

### 2.6. Data Synthesis

Given the heterogeneity in study populations, lipidomic techniques, and outcome measures, a meta-analysis was not performed. Instead, findings were synthesized narratively, with particular attention to patterns of lipid dysregulation across different pathogens, biological plausibility of identified lipids, and consistency of results across studies.

## 3. Results

### 3.1. Study Characteristics and Analytical Platforms

The included studies [12,13,15,18,19,20,21,22,23,24,25,26,27,28,29,30] span diverse geographic regions and clinical settings, with cohorts recruited from Spain, China, Germany, France, Greece, the Netherlands, the United States, and Brazil. Study designs encompassed prospective observational cohorts, retrospective post hoc analyses, cross-sectional investigations, and pilot studies. Patient populations covered a wide clinical spectrum, including adults and children, hospitalized and ICU patients, and individuals with varying degrees of severity across conditions such as CAP, COVID-19, VAP, sepsis, and tuberculosis. Sampling strategies were heterogeneous, drawing on serum, plasma, BALF, tracheal aspirates, urine, nasopharyngeal swabs, and isolated immune cells. Several studies incorporated validation cohorts (e.g., CAPSOD and EARLI) or longitudinal sampling (e.g., Days 1, 3, 6, 12). Most employed untargeted lipidomic approaches on LC-MS/MS or UHPLC-HRMS platforms, ensuring broad coverage of lipid classes, while others adopted targeted analyses for specific phospholipid ratios. Notably, integrative multi-omics frameworks combining lipidomics with proteomics, transcriptomics, or metabolomics provided additional mechanistic insights into host–pathogen interactions. Summary of the studies’ characteristics is displayed in Table 1.

### 3.2. Identified Lipid Biomarkers in LRTIs

PCs and LPCs were the most frequently investigated lipid classes, with multiple studies reporting decreased LPC and variable PC levels in patients with CAP and COVID-19. Notably, specific phosphatidylcholine species such as PC(18:2_20:4), PC(36:4), and PC(38:6) were found to be significantly reduced in severe CAP and were independently associated with disease severity, oxygen requirements, and procalcitonin levels. In particular, PC(18:2_20:4) demonstrated high diagnostic accuracy with an area under the curve (AUC) of 0.954 [13].

Similarly, decreased LPC(22:6-sn2) and increased PC(36:1) were among the most discriminatory lipid alterations in hospitalized COVID-19 patients, with the LPC/PC ratio achieving an AUC > 0.95 for disease differentiation [18]. Other studies reported that the PC/LPC and phosphatidylethanolamine/lysophosphatidylethanolamine (PE/LPE) ratios were positively correlated with systemic inflammation markers and predictive of mortality [22].

Sphingolipids, particularly sphingomyelins and ceramides, were also frequently altered. Sphingomyelin (SM) (34:1) and PC(O-34:1) emerged as strong predictive biomarkers for VAP in COVID-19-associated ARDS, with area under the receiver operating characteristic curve (AUROC) values of 0.85 and 0.83, respectively [21]. In a complementary study, Schuurman et al. (2024) [23] identified significant upregulation of the sphingosine-1-phosphate (S1P) signaling pathway and its regulatory enzymes—sphingosine kinase 1 (SPHK1), UDP-glucose ceramide glucosyltransferase (UGCG), and sphingomyelin phosphodiesterase 1 (SMPD1)—in monocytes and neutrophils of patients with CAP, thereby linking sphingolipid metabolic alterations to pro-inflammatory immune responses.

In terms of fatty acid-derived lipids, oxylipins such as 9- and 13-hydroxyoctadecadienoic acid (9/13-HODE), 15-hydroxyeicosatetraenoic acid (15-HETE), 12,13-epoxyoctadecenoic acid (12,13-EpOME) and 9,10-dihydroxyoctadecenoic acid (9,10-DiHOME) were differentially expressed across viral and bacterial etiologies. Viral CAP was characterized by elevated oxylipins and FA 18:2–containing triglycerides (TGs) and diacylglycerols (DGs), while bacterial CAP showed enrichment in PCs and PC ethers [12].

Several studies identified cholesterol esters as inversely correlated with severity scores and systemic inflammation. In the study by Chouchane et al. [19], a 10% increase in cholesteryl ester (CE) levels by Day 4 in the ICU was associated with a reduced 30-day mortality [odds ration (OR) = 0.84], supporting their prognostic significance.

Notably, lipidomic analyses of tracheal aspirates revealed local lipid remodeling at the infection site. In patients with high VAP suspicion, multiple PC and SM species were upregulated, and these lipid signatures correlated with pathogen type and inflammation [15].

Humes et al. [24] showed that acylcarnitines, DGs, and LPCs defined pathogen-specific clusters, with high TG/DG profiles associated with influenza A H3 and low-lipid profiles with rhinovirus. Zheng et al. [25] reported >10-fold increases in lactosylceramides in CAP, while decreased sphingosine and lysolipids correlated with severity. In tuberculosis, Sun et al. [26] identified bile acid and fatty acid pathway dysregulation, with Angiotensin IV achieving diagnostic AUCs > 0.99. Zhang et al. [27] highlighted three lipid species (CER(24:0) H, HCER(d18:0/22:0) H, PE(18:1/18:1)) as strong TB classifiers. Chen et al. [28] found perturbations in lysoPE, LPC, and PI as discriminators of pneumonia severity in children. Tomalka et al. [29] demonstrated that AA, EPA, DHA, and DPA correlated strongly with WHO COVID-19 severity scores. De Almeida et al. [30] identified key lipid classes (PS, PI, PA vs. PC, SM, DG) that, combined with proteomic profiles, achieved >95% accuracy in COVID-19 diagnosis.

Table 2 displays the main lipid biomarkers identified across studies, their putative biological functions in the context of respiratory infections, and their reported diagnostic or prognostic performance.

### 3.3. Role of Lipidomics in Pathophysiological Insights

One of the most consistent findings across studies is the dysregulation of phospholipids, particularly PCs and LPCs, which are integral to membrane integrity, surfactant composition, and inflammatory signaling. Reduced LPC levels, frequently reported in severe CAP and COVID-19, may reflect increased consumption during acute-phase inflammation and impaired resolution of infection [13,18,19,20]. Furthermore, the altered PC/LPC and PE/LPE ratios, which correlated with pro-inflammatory cytokines, CRP levels, and disease severity, suggest that lipid remodeling enzymes such as lysophosphatidylcholine acyltransferase 1/2 (LPCAT1/2) are tightly regulated in infection-induced stress responses [22].

Lipidomics has also shed light on bioactive lipid mediators such as oxylipins, which include hydroxyoctadecadienoic acids (HODEs) and epoxyoctadecenoic acids (EpOMEs). These metabolites, derived from linoleic acid and other polyunsaturated fatty acids via enzymatic oxidation, are known to modulate vascular permeability, leukocyte recruitment, and immune cell activation. Their reduction or elevation in viral versus bacterial infections points to divergent lipid signaling pathways depending on the pathogenic trigger [12,18].

Another key contribution of lipidomics is the elucidation of sphingolipid metabolism, particularly the role of SMs and S1P in immune regulation. Sphingolipids are not only structural membrane components but also active regulators of neutrophil migration, cytokine release, and lymphocyte trafficking. In CAP patients, upregulation of SPHK1, the enzyme responsible for S1P synthesis, was linked to enhanced monocyte and neutrophil activation, as confirmed by transcriptomic integration and functional inhibition assays [23].

Elevated acylcarnitines indicate disrupted mitochondrial β-oxidation and energy stress, while increased circulating triglycerides—particularly in viral CAP and COVID-19—are consistent with hepatic lipogenesis driven by inflammatory cytokines such as interleukin -6 (IL-6) and tumor necrosis factor alpha (TNF-α) [12,18].

Lipidomic signatures derived from localized samples such as tracheal aspirates further illuminate tissue-specific processes. In VAP, differential expression of surfactant-associated lipids and ether-linked phospholipids pointed to alveolar epithelial injury rather than direct bacterial lipid contribution. These changes were correlated with pathogen-specific profiles, offering insights into pulmonary lipid metabolism at the site of infection [15,21].

These studies further illuminate disease mechanisms. Humes et al. [24] demonstrated that sputum lipid clusters reflected viral subtype–specific metabolic remodeling. Zheng et al. [25] linked BALF lipid patterns with immune cell infiltration, suggesting a role in local host–pathogen interactions. In tuberculosis, Sun et al. [26] and Zhang et al. [27] both implicated bile acid and sphingolipid pathways, consistent with chronic inflammation and immune evasion. Chen et al. [28] showed that *Mycoplasma pneumoniae* infection perturbed lysophospholipid and PI metabolism, reflecting membrane damage. Tomalka et al. [29] revealed that pro-resolving mediators and eicosanoid precursors not only tracked severity but also mapped to interferon suppression in COVID-19. De Almeida et al. [30] demonstrated that systemic lipid and protein signatures serve as robust diagnostic fingerprints of infection.

### 3.4. Lipidomic Alterations by Pathogen Type

In the prospective cohort study by Rischke et al. [12], lipidomic profiling of patients with viral versus bacterial CAP revealed substantial differences. Bacterial CAP was characterized by elevated levels of PCs and ether-linked PCs, consistent with enhanced membrane biosynthesis and neutrophilic activation. In contrast, viral CAP showed increased concentrations of TGs and DGs, particularly those containing linoleic acid (18:2), as well as higher levels of pro-inflammatory oxylipins, including 12,13-EpOME and 9,10-DiHOME.

Similarly, in a pediatric ICU cohort, Virgiliou et al. [15] demonstrated that lipidomic profiles in VAP varied by pathogen. Blood lipid signatures—including specific PCs, SMs, and TGs—differentiated infections due to *Staphylococcus aureus* and *Klebsiella pneumoniae*, supporting the concept of pathogen-specific metabolic remodeling in critically ill children. Notably, PCs (e.g., PC 32:2) and TGs (e.g., TG 48:3) showed good discriminatory performance (AUC > 0.75).

Further evidence for pathogen-specific lipid responses was observed in the study by Kassa-Sombo et al. [21], which examined tracheal aspirate lipidomes in COVID-19 ARDS patients with or without VAP. While not explicitly focused on microbial taxonomy, the study identified eight significantly upregulated lipids [including SM(34:1) and PC(O-34:1)] in VAP patients. These lipids were associated with epithelial cell membrane breakdown rather than direct bacterial lipid production, but their expression patterns reflected the presence and severity of superimposed bacterial infection.

Humes et al. [24] provided clear evidence of pathogen-specific signatures among respiratory viruses, with influenza A H3 enriched in TG/DG and rhinovirus in lipid-depleted profiles. Zheng et al. [25] highlighted CAP-specific increases in lactosylceramides compared to controls. In tuberculosis, both Sun et al. [26] and Zhang et al. [27] revealed distinct lipid dysregulation patterns compared to healthy individuals. Chen et al. [28] distinguished M. pneumoniae pneumonia from bronchitis through plasma/urine metabolite panels. Tomalka et al. [29] observed conserved lipid mediator dysregulation across SARS-CoV-2 variants, while de Almeida et al. [30] identified COVID-19–specific lipid fingerprints distinct from seronegative controls.

### 3.5. Temporal Dynamics and Prognostic Implications

In a multicenter study of critically ill CAP patients, Chouchane et al. [19] documented time-dependent recovery of specific lipid classes, most notably CE and LPC. More than half (58%) of all quantified lipid species were decreased upon ICU admission. However, restoration of CE levels by Day 4 was independently associated with improved outcomes, with a 10% increase corresponding to a 16% reduction in 30-day mortality risk (OR = 0.84).

Similarly, Schuurman et al. [23] assessed monocyte and neutrophil lipidomes at baseline and one month following CAP. While lipid perturbations in monocytes largely resolved during convalescence, neutrophils exhibited sustained alterations, including persistent elevations in polyunsaturated TGs and DGs.

In pediatric populations, Virgiliou et al. [15] conducted serial lipidomic analyses in patients with suspected VAP across four timepoints (Days 1, 3, 6, and 12). A gradual increase in PCs and TGs was observed in the high-suspicion group, parallel with microbiological confirmation and clinical deterioration. Specific lipids such as TG(48:3) and SM(40:1) showed increasing discrimination power over time (AUC > 0.75).

Prognostically, several studies reported lipid-based models that outperformed traditional clinical scores. In the study by Chen et al. [13], phosphatidylcholine species PC(18:2_20:4) and PC(38:6) achieved AUCs of 0.954 and 0.959, respectively, for predicting CAP severity—superior to CURB-65 and the PSI. Likewise, Ma et al. [22] identified that elevated PC/LPC and PE/LPE ratios were positively associated with disease severity, inflammatory markers, and prolonged hospital stay, and independently predicted 30-day mortality (AUC = 0.838 for PC/LPC ratio).

Longitudinal patterns were also evident. Zheng et al. [25] linked BALF lipid clusters to CAP severity, with certain fatty acids discriminating severe vs. non-severe disease. Sun et al. [26] validated lipid-driven TB signatures across training and validation cohorts with very high reproducibility. Chen et al. [28] demonstrated that plasma metabolite panels predicted not only infection vs. health but also disease phenotype, correlating with neutrophil–lymphocyte ratios. Tomalka et al. [29] showed that eicosanoid precursors correlated with WHO severity scores and innate immune suppression, suggesting prognostic utility. De Almeida et al. [30] reported near-perfect reproducibility of lipidomic/proteomic classifiers across independent patient sets.

### 3.6. Integrative Models and Clinical Translation

Several included studies employed multivariate statistical and machine learning techniques to build predictive models based on lipidomic features. In a retrospective cohort of hospitalized COVID-19 patients, Castané et al. [18] used Monte Carlo simulations, principal component analysis (PCA), and partial least squares discriminant analysis (PLS-DA) to identify a panel of discriminatory lipids, including LPC(22:6-sn2) and PC(36:1). The derived ratio LPC(22:6-sn2)/PC(36:1) yielded an AUC exceeding 0.95 for distinguishing COVID-19 from other infectious or inflammatory conditions.

Similarly, Chen et al. [13] developed a ROC-based classifier using individual phosphatidylcholines (e.g., PC(38:6) and PC(18:2_20:4)) that outperformed traditional clinical severity scores such as CURB-65 and PSI, with AUCs > 0.95. These models were not only statistically robust but also biologically interpretable, as the lipid species selected were implicated in inflammation, cellular stress, and membrane dynamics.

The integration of lipidomics with other omics platforms further enhanced discriminatory power and mechanistic understanding. Rischke et al. [12] combined lipidomics with proteomics and metabolomics to differentiate viral from bacterial CAP, demonstrating that pathogen-specific clusters could be generated based on co-expression networks of linoleic acid–derived oxylipins, triglycerides, and host immune markers such as TNF-related apoptosis-inducing ligand (TRAIL) and Lymphocyte-activation gene 3 (LAG-3). Such multi-omic integration supports not only diagnostic classification but also insights into immune-metabolic crosstalk.

Machine learning approaches further advanced translation. Humes et al. [24] applied Bayesian regression and multinomial models to identify pathogen-specific sputum clusters. Zheng et al. [25] used clustering and ROC analyses to link lipid subsets to CAP severity. Sun et al. [26] leveraged multiple algorithms (LASSO, Random Forest, XGBoost) to derive reproducible TB classifiers. Chen et al. [28] developed a 13-metabolite panel (AUC = 0.927–1.0) for pediatric infection stratification. Tomalka et al. [29] combined lipidomics with RNA-seq to map lipid-mediated immune dysregulation in COVID-19. De Almeida et al. [30] integrated serum lipidomics with MALDI-based proteomics, achieving >98% diagnostic accuracy and near-zero false negatives in COVID-19 screening.

Table 3 displays lipidomics findings by clinical category.

## 4. Discussion

This systematic review synthesized evidence from nine original studies that employed lipidomic analyses in the context of LRTIs, including CAP, VAP, and COVID-19. Across diverse populations, analytical platforms, and sample types, a consistent pattern of lipid alterations emerged. Dysregulation of PCs and LPCs was the most commonly reported finding, with decreased LPC levels frequently associated with severe disease. Changes in lipid profiles also demonstrated pathogen-specific patterns, distinguishing viral from bacterial etiologies. Furthermore, temporal dynamics of lipid species were linked to clinical outcomes, with early normalization of certain lipid classes (e.g., cholesterol esters) associated with reduced mortality. Importantly, lipidomic biomarkers often outperformed conventional clinical scores in predictive models, underscoring their potential clinical utility.

Lipidomic analyses have provided critical mechanistic insights into how LRTIs disrupt host lipid metabolism. One of the most consistent findings across studies is the downregulation of LPCs and the alteration of PC/LPC and PE/LPE ratios, particularly in severe CAP and COVID-19 cases. These changes suggest involvement of phospholipid remodeling pathways regulated by LPCAT1/2, enzymes known to be modulated in response to inflammatory stress, potentially reflecting the host’s attempt to maintain membrane integrity and immune regulation [13,18,19,22].

Oxylipins, which are oxygenated derivatives of polyunsaturated fatty acids, were also differentially expressed across pathogen types. Elevated concentrations of 12,13-EpOME and 9,10-DiHOME in viral CAP highlight increased cytochrome P450 and epoxide hydrolase activity, which may mediate pro-inflammatory or vasoactive responses. Conversely, reduced levels of 9/13-HODE and 15-HETE observed in bacterial pneumonia may reflect suppressed resolution-phase signaling and altered lipid mediator balance [12,18].

Additionally, sphingolipid metabolism has emerged as a key regulatory axis in the host response to LRTIs. The upregulation of S1P signaling and its biosynthetic enzyme SPHK1, along with changes in specific SM species (e.g., SM 34:1), suggest a mechanistic link to monocyte and neutrophil activation, as well as cytokine production [21,23]. These alterations likely contribute to broader immunometabolic rewiring and may perpetuate inflammation or immune exhaustion during infection.

Finally, multiple studies reported elevated acylcarnitines and TGs in patients with viral pneumonia, indicating disrupted mitochondrial β-oxidation and enhanced hepatic lipogenesis, respectively—hallmarks of systemic metabolic stress and energy imbalance during acute infection [12,18].

Recent evidence highlights the role of sphingosine-1-phosphate (S1P) signaling in stabilizing the alveolar endothelial–epithelial barrier. Activation of S1PR1 promotes actin cytoskeleton remodeling and adherens-junction assembly through Rac1/Cdc42 signaling, thereby reducing vascular permeability, while S1PR2 activation exerts opposing barrier-disruptive effects [31].

Epithelial damage markers could further contextualize the present findings. Circulating sRAGE reflects type I alveolar epithelial injury and predicts worse outcomes in ARDS [32]. Surfactant protein D (SP-D) is a sensitive marker of type II pneumocyte injury [33], while KL-6/MUC1 is released from regenerating pneumocytes and is correlated with severity of epithelial damage [34]. More recent data suggest that lung tissue expression of RAGE and elafin, but not SP-D, is closely related to histopathological injury severity [35].

Induction of pro-inflammatory cytokines such as TNF-α, IL-1β, IL-6, IL-8, and CCL2 has profound effects on lipid signaling. These cytokines activate sPLA_2_, COX, and LOX pathways, leading to oxylipin generation [36]. In parallel, inflammatory stress increases ceramide accumulation, which promotes alveolar leaks and apoptosis [37].

Despite the promising results, several technical limitations warrant consideration. First, there is significant heterogeneity in analytical platforms (e.g., LC-MS/MS, UHPLC-HRMS, NMR), lipid extraction protocols, and bioinformatic pipelines, which limits direct comparison across studies. Second, many studies utilized untargeted lipidomics, which, while comprehensive, may suffer from challenges in lipid identification, quantification, and inter-laboratory reproducibility. Additionally, normalization techniques, internal standards, and statistical thresholds varied widely across studies, potentially influencing results.

Sample types and timing also varied considerably—ranging from serum to tracheal aspirates and from single to multi-timepoint sampling—which complicates harmonization. Finally, few studies validated their findings in independent cohorts, and even fewer integrated lipidomic data with clinical decision-making tools or prospective outcome measures.

This systematic review has several limitations that should be acknowledged. First, the number of eligible studies was relatively small (n = 9), reflecting the early and still emerging nature of lipidomic applications in LRTIs. As a result, some conclusions are based on limited data, and findings may not be generalizable across all LRTI subtypes, geographic regions, or patient populations. Second, the heterogeneity in study designs, sample types, disease severity, and lipidomic platforms precluded quantitative synthesis through meta-analysis. Variations in analytical methods—including differences in mass spectrometry instrumentation, lipid extraction protocols, normalization strategies, and data processing workflows—introduce methodological variability that complicates direct comparisons across studies.

Third, while most studies achieved high scores on the Newcastle–Ottawa Scale, many lacked external validation cohorts, used small sample sizes, or did not control for potential confounders such as comorbidities, medication use, or nutritional status, all of which can influence the lipidome. Beyond technical heterogeneity, methodological limitations of the included studies should also be considered. Several cohorts may have been subject to selection bias, as recruitment was often restricted to hospitalized or ICU patients, thereby limiting generalizability to milder disease. Moreover, potential confounders such as comorbidities, medication use (e.g., statins, corticosteroids), nutritional status, and prior infections were not consistently controlled for, despite their known influence on the circulating lipidome. These factors may have impacted both the validity and reproducibility of lipidomic associations reported. Future studies should therefore incorporate rigorous adjustment for such confounders and aim to stratify analyses by comorbidity burden and baseline characteristics.

Additionally, few studies conducted longitudinal sampling or integrated lipidomic data with real-time clinical decision-making, limiting insights into the temporal utility of lipid biomarkers in routine care. Fourth, publication bias may have influenced the findings, as studies with null or non-significant results may be underrepresented in the published literature. Moreover, only English-language articles were included, potentially excluding relevant non-English studies. Finally, while this review included both adult and pediatric populations, age-specific differences in lipid metabolism were not separately analyzed due to the limited number of pediatric studies, which may have masked age-related variability in lipidomic signatures.

Despite these limitations, this review provides the first comprehensive synthesis of the current evidence on the role of lipidomics in LRTIs. Future studies addressing the above limitations are needed to validate and expand upon these preliminary findings.

Several gaps remain in the current literature. Most notably, large-scale, multi-center studies are lacking, and many of the available data are derived from pilot or single-center cohorts with modest sample sizes. Future research should prioritize validation of lipid-based biomarkers in diverse patient populations and clinical settings. There is also a need for standardized protocols and reporting frameworks for lipidomic studies, akin to the Minimum Information About a Metabolomics Experiment (MIAME) guidelines used in transcriptomics.

Moreover, integrative multi-omics approaches—including transcriptomics, proteomics, and metabolomics—should be employed more consistently to contextualize lipidomic changes within broader biological networks. Functional studies exploring how specific lipid mediators (e.g., oxylipins, sphingolipids) influence immune cell behavior during infection would also enhance mechanistic understanding.

Finally, efforts should be directed toward translating lipidomic findings into clinically applicable tools, such as rapid point-of-care tests or algorithm-based risk stratification models. Collaboration between clinicians, analytical chemists, and computational biologists will be critical to realizing the translational potential of lipidomics in LRTIs.

## 5. Conclusions

This systematic review highlights the emerging role of lipidomics in unraveling the complex host response to respiratory tract infections. Across diverse clinical settings and infection types, lipidomic profiling has revealed consistent perturbations in key lipid classes—particularly phospholipids, sphingolipids, oxylipins, and acylcarnitines—reflecting alterations in membrane remodeling, immune activation, and systemic metabolic stress. The diagnostic and prognostic potential of lipid-based biomarkers, including specific lipid ratios and composite classifiers, appears promising and, in some studies, exceeds the predictive performance of conventional clinical scoring systems. Moreover, the integration of lipidomics with proteomic and transcriptomic data offers mechanistic insights into pathogen-specific immune pathways and points toward precision medicine approaches in infectious disease management. Nevertheless, substantial methodological heterogeneity, limited validation cohorts, and the lack of standardization across platforms currently constrain clinical translation. Future research should focus on multi-center, longitudinal studies with standardized protocols, age- and pathogen-stratified analyses, and integration with other omics technologies. Overall, lipidomics holds substantial potential not only as a tool for biomarker discovery but also for advancing our understanding of host–pathogen interactions in LRTIs. To bridge discovery with practice, translational studies are urgently required, including prospective clinical trials that test the integration of lipid panels into clinical decision-making algorithms for diagnosis, risk stratification, and treatment guidance. Such efforts will determine whether lipidomics can move beyond biomarker discovery toward actionable tools in real-world patient management.

## Figures and Tables

**Table 1:** Summary of the studies’ characteristics.

First Author	Year	Country	Study Design	Population	Sample Type	Analytical Platform	Lipid Classes Analyzed	Main Findings	NOS
Castané [18]	2022	Spain	Retrospective post hoc cohort study	126 hospitalized COVID-19 patients, 45 hospitalized COVID-19-negative patients (infectious/inflammatory), 50 healthy controls	Serum	Semi-targeted lipidomics by UHPLC-QTOF-MS, machine learning (Monte Carlo, PCA, PLS-DA)	Acylcarnitines, lysophospholipids (LPC, LPE), phosphatidylcholines (PC), oxylipins (e.g., 9/13-HODE, 15-HETE), bile acids, long-chain TGs	COVID-19 and non-COVID inflammatory patients shared a common lipid signature characterized by elevated acylcarnitines and LPE, and decreased 9/13-HODE and 15-HETE. However, specific discrimination between COVID-19 and other conditions was achieved via decreased LPC(22:6-sn2), increased PC(36:1), and changes in secondary bile acids. Arachidonic acid levels were markedly decreased in both COVID-19 and other infectious groups. Machine learning identified oxylipins, carnitines, and phospholipids as key discriminatory features. Alterations in β-oxidation and fatty acid metabolism were prominent. The ratio of LPC22:6-sn2/PC36:1 achieved an AUC > 0.95. No association was found with ICU admission or mortality.	8/9
Chen [13]	2021	China	Cross-sectional pilot study	28 CAP patients (13 severe, 15 non-severe), 20 matched non-CAP controls	Serum	Untargeted UHPLC-MS/MS lipidomics, OPLS-DA, ROC, multivariate regression	Glycerophospholipids (PC, PE), sphingolipids (SM, HexCer), lysophospholipids (LPC, LPE), diacylglycerols, cholesterol esters, free fatty acids	Lipid profiles differed significantly across NC, NSCAP, and SCAP groups. CAP patients exhibited reduced LPC and PE levels, increased Hex2Cer and cholesterol esters. Four lipids (PC[16:0_18:1], PC[18:2_20:4], PC[36:4], PC[38:6]) outperformed CURB-65 and PSI in ROC analysis for disease severity. PC(18:2_20:4) had AUC 0.954, PC(38:6) had AUC 0.959. PC(18:2_20:4) and PC(36:4) correlated negatively with FiO2 and PCT; PC(16:0_18:1) positively with PCT. Lower PC levels were linked to longer hospital stay and higher 30-day mortality. Combined phospholipid biomarkers showed potential for disease monitoring, diagnosis, and prognosis in CAP.	7/9
Chouchane [19]	2024	Netherlands	Prospective cohort study	169 ICU patients with CAP sepsis, 51 noninfected ICU controls, 48 outpatient controls; plus two validation cohorts (CAPSOD, EARLI)	Plasma	Untargeted lipidomics via HPLC-MS (1833 lipid species across 33 classes), data validated in external cohorts	Cholesterol esters, triacylglycerols, phospholipids (PC, PE, LPC), sphingomyelins, ceramides, sulfatides, plasmalogens, lysolipids	Patients with sepsis due to CAP exhibited a profound shift in the plasma lipidome compared to both healthy and noninfected ICU controls. 58% of lipid species were decreased, while 6% increased. Cholesterol esters and lysophospholipids showed strong inverse associations with SOFA score and systemic inflammation. Recovery of specific lipids such as cholesterol esters by Day 4 in ICU was linked to lower 30-day mortality (e.g., OR = 0.84 per 10% increase). The lipidomic profile showed partial recovery over time, and a specific TG-rich pattern distinguished CAP from other ICU patients. LPC and Chol-E emerged as key prognostic lipids. Lipid class patterns were validated across CAPSOD and EARLI cohorts. Results support lipidomics as a biomarker and prognostic tool in CAP-associated sepsis.	9/9
Rischke [12]	2025	Germany	Prospective cohort study	69 patients with community-acquired pneumonia (CAP): 43 viral (incl. COVID-19), 26 bacterial	Plasma (baseline, day 3, day 7)	LC-MS/MS (MxP Quant 500), Olink proteomics, lipid network enrichment (LINEX2), PLS-DA, PCA	Phosphatidylcholines, ether-PCs, lysophosphatidylcholines (LPC), triglycerides, diglycerides, bile acids (GCA, TCA, TCDCA), oxylipins (EpOME, DiHOME)	Distinct plasma lipidomic profiles differentiated viral from bacterial CAP. Bacterial CAP was characterized by elevated PCs and PC-ethers, while viral CAP showed increased TGs, DGs (especially FA 18:2-containing), and linoleic acid–derived oxylipins (12,13-EpOME, 9,10-DiHOME). Proteomic markers like TRAIL, LAG-3, and LAMP3 were elevated in viral CAP, while CLEC4D and EN-RAGE were elevated in bacterial CAP. Integrated clustering of lipidomic, metabolomic, and proteomic analytes supported co-expression of pathogen-specific patterns. PLS-DA and hierarchical clustering identified robust discriminatory features. Findings indicate the potential of lipidomics and multi-omics in pathogen-specific diagnosis and individualized treatment strategies in CAP.	8/9
Saballs [20]	2024	Spain	Prospective observational study	71 CAP patients, 75 COVID-19 pneumonia patients, 75 healthy controls (age- and sex-matched)	Serum	1H NMR spectroscopy (Liposcale^®^ assay), BUME extraction, LipSpin, multivariate analysis (PLS-DA, random forest, ROC)	Phosphatidylcholine, lysophosphatidylcholine, PUFA, DHA, esterified/free cholesterol, triglycerides, HDL/LDL/VLDL subclasses, glycoproteins	Both CAP and COVID-19 pneumonia patients exhibited hypolipidemia with reduced levels of HDL-c, phosphatidylcholine, lysophosphatidylcholine, PUFA, and DHA. Severity was associated with increased VLDL-c, IDL-c, LDL-tg/LDL-c, triglycerides, and glycoproteins (GlycA, GlycB, GlycF), along with decreased HDL particles and esterified cholesterol. COVID-19 patients showed more pronounced alterations. A lipidomic-metabolomic model based on PC, glycerophospholipids, creatine, glutamate, isoleucine, alanine, and glycoproteins achieved AUC = 0.935 for etiology classification and 0.931 for severity. Metabolites linked to inflammation and energy metabolism (lactate, glucose, creatine, BCAAs, glutamate) were elevated. These alterations reflect lipid-mediated immune modulation, metabolic reprogramming, and potential for diagnostic/prognostic biomarker development.	7/9
Kassa-Sombo [21]	2025	France	Prospective observational cohort study	39 patients with COVID-19 ARDS (26 with VAP, 13 without VAP), matched controls	Tracheal aspirate	Untargeted lipidomics via UHPLC-HRMS (Q-Exactive MS), data processed with Workflow4Metabolomics/XCMS	Phosphatidylcholines, phosphatidylethanolamines, sphingomyelins, ether-linked PCs and PEs, lysophospholipids	Significant alterations in the tracheal aspirate lipidome were observed in VAP versus non-VAP COVID-19 ARDS patients (*p* = 0.003). Among 272 identified lipids, PCs were most frequently dysregulated, with 17 upregulated and 6 downregulated. SM(34:1) and PC(O-34:1) were the most predictive biomarkers for VAP, showing AUROC of 0.85 and 0.83, respectively. Eight key lipids were identified via multivariate analyses (PCA, PLS-DA, OPLS-DA), all upregulated in VAP. Combined lipid biomarkers modestly improved diagnostic performance (AUROC up to 0.86). These results suggest that lipidomics of tracheal aspirates offers potential for accurate VAP diagnostics and highlights lipid remodeling at the site of infection.	8/9
Ma [22]	2022	China	Prospective multi-center cohort study	58 patients with CAP (30 NSCAP, 28 SCAP), 11 healthy controls	Serum	Targeted LC-MS/MS, qRT-PCR validation, GEO database transcriptome analysis	Phosphatidylcholine (PC), lysophosphatidylcholine (LPC), phosphatidylethanolamine (PE), lysophosphatidylethanolamine (LPE)	LPC levels were decreased, while PE, PC, PC/LPC, and PE/LPE ratios were increased in CAP. PE combined with CURB-65 predicted severity (AUC = 0.848), while PC/LPC ratio improved 30-day mortality prediction (AUC = 0.838). PC(36:4) and LPC(18:2/0:0) emerged as species-level biomarkers. Expression of LPCAT2 was upregulated and LPCAT1 downregulated in SCAP; LPCAT2 positively correlated with inflammatory genes, LPCAT1 negatively. Lipid ratios (PC/LPC, PE/LPE) and PE were positively correlated with CRP, neutrophil percentage, and PSI, and negatively with albumin and lymphocytes. Alterations in Lands cycle enzymes support metabolic dysregulation during infection.	8/9
Schuurman [23]	2024	Netherlands	Case–control cohort study	48 CAP patients (baseline and 1-month follow-up), 25 matched noninfectious controls	Isolated blood monocytes and neutrophils	Untargeted HPLC-MS lipidomics with transcriptomics integration	Sphingolipids (SM, ceramides, S1P), phospholipids (PC, PE), lysophospholipids (LPC, LPE), fatty acids, diacylglycerols (DG), triglycerides (TG), BMPs	Pneumonia significantly altered the lipidomic landscape of monocytes and neutrophils, with distinct profiles. Monocyte lipid changes were mostly decreases in PC, PE, and SM species and resolved after 1 month. In contrast, neutrophils showed persistent changes with increased PC, PE, DG, BMP, and polyunsaturated TGs. Sphingolipid metabolism, particularly S1P signaling, was upregulated. Transcriptomic analysis confirmed upregulation of key enzymes (SPHK1, UGCG, SMPD1). Functional validation showed that inhibiting SPT and SPHK1 blunted cytokine production in both cell types. The study demonstrated a mechanistic link between altered lipid profiles and immune function during CAP.	9/9
Virgiliou [15]	2024	Greece	Prospective pilot cohort study	20 critically ill pediatric patients (12 high VAP suspicion, 8 low suspicion)	Plasma (blood samples at 4 timepoints: Days 1, 3, 6, 12)	Untargeted LC-HRMS (UPLC-TIMS-TOF/MS), multivariate + univariate analysis, MS-DIAL, Lipostar2	Phosphatidylcholines (PC), lysophosphatidylcholines (LPC), sphingomyelins (SM), triglycerides (TG), diglycerides (DG), cholesterol esters (CE), carnitines	Untargeted lipidomics revealed 144 blood lipid species in critically ill pediatric patients with VAP suspicion. PCs, SMs, TGs, and DGs were significantly altered between high and low mCPIS groups. High suspicion group showed increased levels of PCs and TGs over time. Discriminatory lipids (e.g., PC 32:2, TG 48:3, SM 40:1) had AUC > 0.75. Specific lipid profiles were associated with culture-confirmed pathogens, including S. aureus and K. pneumoniae. Multivariate models (OPLS-DA) distinguished both VAP severity and pathogen type. Phospholipid shifts correlated with inflammatory markers and may indicate pathogen-specific metabolic remodeling. The study supports lipidomics as a promising diagnostic tool for early VAP identification and microbial stratification in pediatric ICU settings.	7/9
Humes [24]	2022	USA	Cross-sectional study	30 patients with confirmed viral respiratory infections: Influenza A H1-2009 (n = 9), Influenza A H3 (n = 11), Rhinovirus (n = 10)	Induced sputum	Untargeted LC–HRMS (Thermo Q-Exactive Orbitrap, UHPLC), Bayesian profile regression, multinomial logistic regression	>600 lipid species across 27 lipid classes (positive mode) and 23 classes (negative mode). Most abundant: TG, PE, PC, SM, ether-PC, ether-PE. Differential lipids included Acylcarnitines (10:1, 16:1, 18:2), DGs (16:0_18:0, 18:0_18:0), LPC (12:0, 20:5), PE (18:0_18:0), TGs (various species).	Three lipid clusters were identified. Cluster 1 (high TG/DG) associated with Influenza A H3, while Cluster 3 (low lipid levels) associated with Rhinovirus. Odds of H3 infection were 15× higher vs. Rhinovirus in lipid-rich cluster (OR 15.0, 95% CI 1.03–218.3; *p* = 0.047). Distinct lipid signatures suggest pathogen-specific metabolic remodeling. Findings support sputum lipidomics as a tool for differentiating viral respiratory infections.	7/9
Zheng [25]	2019	China	Multicenter observational cohort (pilot)	52 patients with community-acquired pneumonia (CAP); 68 controls (35 healthy, 33 with non-infectious lung disease); bronchoscopy performed within 72 h of admission; 30-day follow-up	BALF	Untargeted lipidomics via HPLC-MS (UHPLC-Q Exactive Orbitrap), PCA, k-means clustering, correlation and ROC analyses	Fatty acids (SFA, MUFA, PUFA), acylcarnitines, sphingolipids (SM, GM3, LacCer, sphingosine), neutral lipids (TG, DG), phospholipids (PC, PE, PI, PG, PS), lysolipids (LPC, LPE, LPG)	CAP patients showed markedly increased lactosylceramides (>10-fold), MUFA/PUFA, PE, and DG (~2-fold), with decreased sphingosine, PG, and lysolipids (LPC, LPE, LPG). Lipid clusters correlated with inflammation and severity: SM(d34:1) inversely with macrophages (adj r = −0.462), PE(18:1p/20:4) positively with PMNs (adj r = 0.541). Certain fatty acids discriminated severe CAP from non-severe (AUC ~0.74–0.76). Findings support BALF lipidomics as a marker of CAP severity and immune response.	8/9
Sun [26]	2025	China	Case–control study with training (72 TB vs. 78 HCs) and validation set (30 TB vs. 30 HCs)	102 active TB patients, 108 healthy controls	Plasma	Untargeted metabolomics and lipidomics by UHPLC-HRMS; machine learning (LASSO, Random Forest, XGBoost)	Broad plasma metabolites including lipids (bile acids, amino acids, phospholipids, oxylipins)	Identified 282 differential metabolites in training set, 214 validated. KEGG enrichment: lipid metabolism pathways. Seven core metabolites (Angiotensin IV, glycochenodeoxycholic acid, methyl indole-3-acetate, dulcitol, Asp-Phe, benzamide, carbadox). Angiotensin IV had very high diagnostic accuracy (AUC = 0.999 training; 0.991 validation; sensitivity 98.6–100%, specificity 96.7–100%). Lipid dysregulation (bile acid metabolism, fatty acid metabolism) central to TB profile.	8/9
Zhang [27]	2025	China	Case–control study	50 newly diagnosed pulmonary TB patients vs. 50 age- and sex-matched healthy controls	Plasma	LC-MS/MS lipidomics (untargeted)	TAGs, ceramides (CER), hexosylceramides (HCER), phosphatidylethanolamines (PE), phosphatidylcholines (PC), total cholesterol, triglycerides, HDL, LDL	PTB patients had significantly lower total cholesterol, triglycerides, HDL, LDL, TAG, CER, and HCER, while PE and PC were significantly elevated (*p* < 0.05). From 633 detected lipids, 61 were differentially expressed. Three lipid species (CER(24:0) H, HCER(d18:0/22:0) H, and PE(18:1/18:1)) demonstrated strong diagnostic potential (AUC > 0.75). Lipid alterations correlated weakly/moderately with age and glucose but not with BMI, sex, or	8/9
Chen [28]	2023	China	Prospective observational study	30 children with *Mycoplasma pneumoniae* infections (17 bronchitis, 13 pneumonia) and 35 healthy controls	Plasma, urine	Untargeted lipidomics (UHPLC-LTQ-Orbitrap XL MS), metabolomics (GC–MS)	Lysophosphatidylethanolamines (LPE), lysophosphatidylcholines (LPC), phosphatidylcholines (PC), phosphatidylethanolamines (PE), phosphatidylinositols (PI), triglycerides (TG)	Identified 14 differential lipids and >50 plasma/urine metabolites associated with infection. Perturbations in lysoPE, LPC, PI, and TG reflected membrane damage and altered energy metabolism. Pathways affected included amino acid, nucleotide, and energy metabolism. A 13-metabolite plasma panel (e.g., L-hydroxyproline, creatine, inosine, citric acid, cystine, glycine) discriminated bronchitis vs. pneumonia with AUC = 0.927 and infection vs. healthy with AUC = 1. Several metabolites correlated with neutrophil/lymphocyte ratios and disease severity.	8/9
Tomalka [29]	2025	USA	Case–control, multi-omics study	10 hospitalized COVID-19 patients, 10 matched healthy controls; extended analysis on 58 NP swab samples (ancestral, Delta, Omicron)	Plasma, PBMCs, nasopharyngeal swabs	GC–MS fatty acid panel, bulk RNA-seq (PBMCs), scRNA-seq (nasopharyngeal)	Fatty acids and eicosanoid precursors (AA, EPA, DHA, DPA, linoleic acid, dihomo-γ-linolenic acid), pro-resolving mediators (lipoxins, resolvins)	COVID-19 patients showed significantly increased AA, EPA, DHA, and DPA, all correlating with WHO disease severity (rho 0.70–0.74). Elevated eicosanoid precursors were linked to severe disease (WHO score > 5). Multi-omics revealed positive correlations of inflammatory lipids (notably EPA) with cell cycle progression, DNA damage, and ER stress pathways in PBMCs. Severe cases had reduced innate immune and interferon signaling. NP swabs confirmed upregulation of lipid mediator synthesis genes (ALOX5, ALOX15, PLA2G4A), especially in goblet and ciliated cells, across SARS-CoV-2 variants. Findings implicate lipid mediators in driving both inflammation and resolution, with potential prognostic/therapeutic value.	8/9
de Almeida [30]	2025	Brazil	Case–control, multiomics diagnostic study	239 serum samples (119 COVID-19 positive, 120 negative by ELISA); additional proteomic analysis on 300 samples (150 positive, 150 negative)	Serum	Direct infusion ESI(±)-Orbitrap MS for lipidomics; MALDI(+)-TOF MS for proteomics; machine learning (PCA, PLS-DA, SVM)	Glycerophospholipids (PC, PE, PS, PI, PG, PA, LPC, LPG, PIP), glycerolipids (DG, MG, TG), sphingolipids (SM), sterol lipids (Chol, CE), fatty acyls	Identified 16 key lipids in negative-ion mode (e.g., PS, sterols, fatty acids) and 18 in positive-ion mode (e.g., LPC, PC, SM, DG). COVID-19 positive patients showed enrichment in PS, PI, and PA, while controls showed higher PCs. SVM models achieved 96.7–100% sensitivity and 82–97% specificity, with accuracies up to 98.9%. Proteomic MALDI-TOF profiles also achieved ~99% accuracy. Demonstrated lipidomic/proteomic spectral signatures as powerful high-throughput COVID-19 diagnostics with near-zero false negatives.	8/9

AA—Arachidonic Acid; AUC—Area Under the Curve; ARDS—Acute Respiratory Distress Syndrome; BCAAs—Branched-Chain Amino Acids; BMP—Bis(monoacylglycerol)phosphate; BUME—Butanol–Methanol Extraction; CAP—Community-Acquired Pneumonia; CE—Cholesteryl Esters; Chol—Cholesterol; COVID-19—Coronavirus Disease 2019; CRP—C-Reactive Protein; CURB-65—Confusion, Urea, Respiratory Rate, Blood pressure, Age ≥65; DHA—Docosahexaenoic Acid; DG—Diacylglycerol; DiHOME—Dihydroxyoctadecenoic Acid; DPA—Docosapentaenoic Acid; EPA—Eicosapentaenoic Acid; EpOME—Epoxyoctadecenoic Acid; FA—Fatty Acid; FiO_2_—Fraction of Inspired Oxygen; GCA—Glycocholic Acid; GEO—Gene Expression Omnibus; GM3—Monosialodihexosylganglioside; HDL—High-Density Lipoprotein; HexCer—Hexosylceramide; HDL-c—High-Density Lipoprotein Cholesterol; HRMS—High-Resolution Mass Spectrometry; ICU—Intensive Care Unit; IDL—Intermediate-Density Lipoprotein; IDL-c—Intermediate-Density Lipoprotein Cholesterol; LacCer—Lactosylceramide; LAG-3—Lymphocyte Activation Gene 3; LAMP3—Lysosome-Associated Membrane Glycoprotein 3; LC-MS/MS—Liquid Chromatography–Tandem Mass Spectrometry; LDL—Low-Density Lipoprotein; LDL-c—Low-Density Lipoprotein Cholesterol; LDL-tg—Low-Density Lipoprotein Triglycerides; LINEX2—Lipid Network Explorer 2; LPC—Lysophosphatidylcholine; LPCAT1/2—Lysophosphatidylcholine Acyltransferase 1/2; LPE –Lysophosphatidylethanolamine; LPG—Lysophosphatidylglycerol; MaCPIS—Modified Clinical Pulmonary Infection Score; MG—Monoglyceride; MS-DIAL—Mass Spectrometry–Data Independent AnaLysis; NMR—Nuclear Magnetic Resonance; NOS—Newcastle–Ottawa Scale; OPLS-DA—Orthogonal Partial Least Squares Discriminant Analysis; PA—Phosphatidic Acid; PCA—Principal Component Analysis; PC—Phosphatidylcholine; PCR—Polymerase Chain Reaction; PE—Phosphatidylethanolamine; PG—Phosphatidylglycerol; PI—Phosphatidylinositol; PIP—Phosphatidylinositol Phosphate(s); PLS-DA—Partial Least Squares Discriminant Analysis; PBMCs—Peripheral Blood Mononuclear Cells; PCT—Procalcitonin; PS—Phosphatidylserine; PSI—Pneumonia Severity Index; PUFA—Polyunsaturated Fatty Acid; qRT-PCR—Quantitative Reverse Transcription Polymerase Chain Reaction; ROC—Receiver Operating Characteristic; SCAP—Severe Community-Acquired Pneumonia; scRNA-seq—Single-Cell RNA Sequencing; SM—Sphingomyelin; SOFA—Sequential Organ Failure Assessment; SPHK1—Sphingosine Kinase 1; S1P—Sphingosine-1-Phosphate; TG—Triglyceride; TIMS-TOF/MS—Trapped Ion Mobility Spectrometry—Time-of-Flight Mass Spectrometry; TRAIL—TNF-Related Apoptosis-Inducing Ligand; UHPLC—Ultra-High Performance Liquid Chromatography; UHPLC-HRMS—Ultra-High Performance Liquid Chromatography—High Resolution Mass Spectrometry; UPLC—Ultra Performance Liquid Chromatography; VAP—Ventilator-Associated Pneumonia; VLDL—Very Low-Density Lipoprotein; XCMS—Cross-platform Chromatographic Alignment and Peak Picking Software.

**Table 2:** Main lipid biomarkers, biological functions, and diagnostic/prognostic performance.

Lipid Biomarker (or Ratio)	Putative Biological Function in LRTIs	Diagnostic/Prognostic Performance (Study)
PC(18:2_20:4), PC(36:4), PC(38:6)	Structural phosphatidylcholines; membrane fluidity; inflammatory remodeling	Lower in severe CAP; correlated with FiO_2_ (–) and PCT; predicted severity better than CURB-65/PSI (AUC 0.954–0.959); lower levels linked to prolonged hospitalization and higher 30-day mortality [13]
PC/LPC and PE/LPE ratios	Reflect Lands’ cycle activity (LPCAT1/2); membrane repair and inflammatory remodeling	Higher in CAP; correlated with CRP, neutrophils, PSI; PC/LPC ratio predicted 30-day mortality (AUC 0.838) and improved CURB-65 for severity [22]
LPC(22:6-sn2)/PC(36:1)	Resolution lipid vs. structural PC; inflammatory discrimination	Discriminated COVID-19 from other infectious/inflammatory admissions (AUC > 0.95) [18]
SM(34:1) and PC(O-34:1)	Sphingomyelin and ether-PC; markers of epithelial/surfactant injury	Best tracheal aspirate predictors of VAP in COVID-19 ARDS (AUROC 0.85 and 0.83), outperforming CRP/PCT [21]
Cholesteryl esters (CE)	Lipid transport; immune/metabolic recovery	Day-4 rise in CE associated with lower 30-day mortality in CAP sepsis (OR 0.84 per 10% increase) [19]
Oxylipins (12,13-EpOME; 9,10-DiHOME)	Linoleic acid-derived mediators; vascular and leukocyte signaling	Enriched in viral vs. bacterial CAP; contributed to pathogen-specific stratification [12]
Lactosylceramides (LacCer)	Sphingolipid signaling; immune activation	>10-fold increase in BALF in CAP; tracked inflammatory cell patterns and severity [25]
Acylcarnitines	Indicators of mitochondrial β-oxidation dysfunction and energy stress	Elevated in COVID-19 and infectious controls; discriminatory in machine-learning models [18,24]
S1P axis (increase in SPHK1)	Neutrophil/monocyte activation; cytokine production	Immune-cell lipidomics showed SPHK1-driven S1P signaling; inhibition reduced cytokine release [23]
NMR glycoproteins (GlycA/B/F)	Markers of systemic inflammation	Higher with pneumonia severity; included in high-AUC etiology/severity models [20]
AA, EPA, DHA, DPA (eicosanoid precursors)	Pro- and pro-resolving mediator pools; immunometabolic reprogramming	Increased with COVID-19 severity (ρ ≈ 0.70–0.74 vs. WHO score) [29]
Specific lipids in TB (e.g., CER(24:0)H, HCER(d18:0/22:0)H, PE(18:1/18:1))	Sphingolipid and PE dysregulation; reflect chronic inflammation and immune evasion	Strong diagnostic classifiers in pulmonary TB (AUC > 0.75) [27]
Mycoplasma pneumoniae perturbations (LPE, LPC, PI, TG)	Reflect membrane damage and altered energy metabolism	Differentiated bronchitis vs. pneumonia and correlated with severity in children [28]

AA—Arachidonic acid; ARDS—Acute Respiratory Distress Syndrom; AUC—Area Under the Receiver Operating Characteristic Curv; BALF—Bronchoalveolar Lavage Fluid; CAP—Community-Acquired Pneumonia; CE—Cholesteryl Ester; CER—Ceramid; CRP—C-Reactive Protein; CURB-65—Clinical severity score for pneumonia (Confusion, Urea, Respiratory rate, Blood pressure, Age ≥ 65 years); DG—Diacylglycerol; DHA—Docosahexaenoic acid; DPA—Docosapentaenoic acid; EPA—Eicosapentaenoic acid; FiO_2_—Fraction of Inspired Oxygen; GlycA/B/F—NMR glycoprotein signals A, B, F; HCER—Hexosylceramide; HDL-c—High-Density Lipoprotein cholesterol; LacCer—Lactosylceramide; LPC—Lysophosphatidylcholine; LPE—Lysophosphatidylethanolamine; mCPIS—Modified Clinical Pulmonary Infection Score; NMR—Nuclear Magnetic Resonance; PC—Phosphatidylcholine; PE—Phosphatidylethanolamine; PI—Phosphatidylinositol; PCT—Procalcitonin; S1P—Sphingosine-1-Phosphate; SM—Sphingomyelin; SOFA—Sequential Organ Failure Assessment; SPHK1—Sphingosine Kinase 1; TB—Tuberculosis; TG—Triacylglycerol; VAP—Ventilator-Associated Pneumonia; WHO score—World Health Organization COVID-19 clinical progression scale.

**Table 3:** Cross-study tabulation of lipidomics findings by clinical category.

Dimension	Bacterial VAP	Bacterial CAP	Viral VAP (Non-COVID-19)	Viral CAP (Non-COVID-19)	COVID-19	Tuberculosis	Mycoplasma Pneumoniae
Representative cohorts and samples	Pediatric ICU VAP suspicion; plasma; serial samples Days 1–12 [15]	CAP cohorts incl. sepsis; serum/plasma; multicenter and ICU [13,19,22]	—	Influenza A, Rhinovirus; sputum [24]	Hospitalized patients; serum/tracheal aspirate [18,20,21,29,30]	Multicenter CAP-like cohorts; plasma [26,27]	Children with M. pneumoniae infections; plasma/urine [28]
Major lipid pattern	PCs, SMs, TGs, DGs altered; higher PCs and TGs in high mCPIS [15]	↑ PCs and ether-PCs; ↓ LPC; CE and LPC inversely tracked SOFA/inflammation; TG-rich signature in sepsis [13,19,22]	—	↑ TGs, DGs, oxylipins in influenza; rhinovirus lipid-depleted [24]	↓ LPC(22:6-sn2), ↑ PC(36:1), acylcarnitines ↑; hypolipidemia with ↓ HDL-c/PC/LPC; ↑ GlycA/B/F [18,20,29,30]	↑ PE, PC; ↓ total cholesterol, TAGs, ceramides; diagnostic lipid classifiers identified [26,27]	Perturbations in LPC, LPE, PI, TG reflecting infection severity [28]
Signature lipids	PC 32:2 ↑, TG 48:3 ↑, SM 40:1 ↑; AUC > 0.75 [15]	PC(16:0_18:1), PC(18:2_20:4), PC(36:4), PC(38:6) ↓ with severity; CE recovery prognostic [13,19,22]	—	Cluster-specific acylcarnitines, DGs, TGs, LPCs [24]	SM(34:1), PC(O-34:1) best VAP markers; LPC(22:6-sn2)/PC(36:1) ratio AUC > 0.95 [18,21]	CER(24:0)H, HCER(d18:0/22:0)H, PE(18:1/18:1) diagnostic [27]	Differential LPE, LPC, PI species [28]
Diagnostic/prognostic readouts	Discriminated VAP severity and pathogen type; AUC > 0.75 for select lipids [15]	PC species outperformed CURB-65/PSI (AUC ~0.95); CE recovery was associated with lower 30-day mortality (OR 0.84) [13,19,22]	—	Influenza A H3 odds 15× higher vs. Rhinovirus in lipid-rich cluster [24]	High diagnostic accuracy (AUC up to 0.95–0.99); lipid panels outperformed CRP/PCT [18,20,21,29,30]	High AUC (>0.75–0.99) for TB classifiers [26,27]	Plasma/urine metabolite-lipid panels AUC 0.927–1.0 [28]
Inflammatory mediators	Lipid shifts correlated with inflammatory markers [15]	Correlated with CRP, PCT, PSI [13,22]	—	Not reported [24]	GlycA/B/F glycoproteins correlated with severity; CRP/PCT comparison [20,21]	Correlation with immune cell profiles, inflammation [26,27]	Correlated with neutrophil/lymphocyte ratio [28]

AUC—Area Under the Receiver Operating Characteristic Curve; BALF—Bronchoalveolar Lavage Fluid; CAP—Community-Acquired Pneumonia; CE—Cholesteryl Ester; CER—Ceramid; CRP—C-Reactive Protein; CURB-65—Clinical severity score for pneumonia (Confusion, Urea, Respiratory rate, Blood pressure, Age ≥65 years); DG—Diacylglycerol; FiO_2_—Fraction of Inspired Oxygen; GlycA/B/F—NMR glycoprotein signals A, B, F; HCER—Hexosylceramide; HDL-c—High-Density Lipoprotein cholesterol; ICU—Intensive Care Unit; LacCer—Lactosylceramide; LPC—Lysophosphatidylcholine; LPE—Lysophosphatidylethanolamine; mCPIS—Modified Clinical Pulmonary Infection Score; NMR—Nuclear Magnetic Resonance; PC—Phosphatidylcholine; PE—Phosphatidylethanolamine; PI—Phosphatidylinositol; PCT—Procalcitonin; SM—Sphingomyelin; SOFA—Sequential Organ Failure Assessment; SPHK1—Sphingosine Kinase; TB—Tuberculosis; TG—Triacylglycerol; VAP—Ventilator-Associated Pneumonia. ↑: increase; ↓ decrease.

## Data Availability

Not applicable, no new data were created or analyzed in this study.
